# Are primary care and continuity of care associated with asthma-related acute outcomes amongst children? A retrospective population-based study

**DOI:** 10.1186/s12875-021-01605-7

**Published:** 2022-01-14

**Authors:** Sarah Cooper, Elham Rahme, Sze Man Tse, Roland Grad, Marc Dorais, Patricia Li

**Affiliations:** 1 Department of Family Medicine, McGill University, Montréal, Québec Canada; 2 Centre for Outcomes Research and Evaluation, Research Institute of the McGill University Health Centre, 5252 Boulevard de Maisonneuve O, Montréal, Québec H4A 3S5 Canada; 3 Department of Medicine, McGill University, Montréal, Québec Canada; 4 Department of Epidemiology, Biostatistics and Occupational Health, McGill University, Montréal, Québec Canada; 5 Department of Pediatrics, Université de Montréal, Montréal, Québec Canada; 6 StatSciences Inc., Notre-Dame-de-l’Île-Perrot, Québec Canada; 7 Department of Pediatrics, McGill University, Montréal, Québec Canada

**Keywords:** Health service research, Children, Asthma, Family medicine, Continuity of care, Primary care access

## Abstract

**Background:**

Having a primary care provider and a continuous relationship may be important for asthma outcomes. In this study, we sought to determine the association between 1) having a usual provider of primary care (UPC) and asthma-related emergency department (ED) visits and hospitalization in Québec children with asthma and 2) UPC continuity of care and asthma outcomes.

**Methods:**

Population-based retrospective cohort study using Québec provincial health administrative data, including children 2-16 years old with asthma (*N* = 39, 341). Exposures and outcomes were measured from 2010-2011 and 2012-2013, respectively. Primary exposure was UPC stratified by the main primary care models in Quebec (team-based Family Medicine Groups, family physicians not in Family Medicine Groups, pediatricians, or no assigned UPC). For those with an assigned UPC the secondary exposure was continuity of care, measured by the UPC Index (high, medium, low). Four multivariate logistic regression models examined associations between exposures and outcomes (ED visits and hospitalizations).

**Results:**

Overall, 17.4% of children had no assigned UPC. Compared to no assigned UPC, having a UPC was associated with decreased asthma-related ED visits (pediatrician Odds Ratio (OR): 0.80, 95% Confidence Interval (CI) [0.73, 0.88]; Family Medicine Groups OR: 0.84, 95% CI [0.75,0.93]; non-Family Medicine Groups OR: 0.92, 95% CI [0.83, 1.02]) and hospital admissions (pediatrician OR: 0.66, 95% CI [0.58, 0.75]; Family Medicine Groups OR: 0.82, 95% CI [0.72, 0.93]; non-Family Medicine Groups OR: 0.76, 95% CI [0.67, 0.87]). Children followed by a pediatrician were more likely to have high continuity of care. Continuity of care was not significantly associated with asthma-related ED visits. Compared to low continuity, medium and high continuity of care decreased asthma-related hospital admissions, but none of these associations were significant.

**Conclusion:**

Having a UPC was associated with reduced asthma-related ED visits and hospital admissions. However, continuity of care was not significantly associated with outcomes. The current study provides ongoing evidence for the importance of primary care in children with asthma.

**Supplementary Information:**

The online version contains supplementary material available at 10.1186/s12875-021-01605-7.

## Background

For children with asthma, primary care physicians may play an essential role in delivering evidence-based management, including assessing asthma control, ensuring appropriate use of medications, providing asthma education and action plans, and referring to asthma specialists when needed [1]. Population-based studies in Canada and the United Kingdom have demonstrated that areas with high compared to low supply of, or access to, primary care physicians reduced the risk of emergency department (ED) visits and hospitalizations for children with asthma [2, 3]. However, few large-scale studies have demonstrated the impact of having a usual provider of primary care (UPC) and continuity with this provider. Continuity of care, a core attribute of primary care [4], is defined as a health care service that extends over some time, where there is a timely and effective exchange of health information between a patient and their individual medical professional or within a medical team [5]. Nearly two decades ago, Christakis et al. demonstrated that for a group of children in the United States enrolled in a large health maintenance organization and another group enrolled in Medicaid, increased continuity was associated with decreased acute health services utilization (ED visits, hospitalizations); the risk was further decreased for children with asthma [6, 7].

In Québec, Canada, children who are residents of the province have access to primary care providers through public health insurance in the form of pediatricians, family physicians who belong to team-based Family Medicine Groups (FMG), and family physicians not part of an FMG [8, 9]. FMGs were implemented as part of primary care reforms since 2002 to improve the delivery of primary care services [9]. To date, there is little evidence to support such alternative primary care models that may improve continuity of care through informational and team-based continuity [9, 10]. To provide evidence to support policies for ongoing efforts to improve access to primary care and interventions for continuity of care, we aimed to determine the association between having a UPC and continuity of care with asthma-related acute outcomes care in a population-based cohort of children with asthma living in Québec, Canada. We hypothesized that having a UPC, and high continuity of care amongst those with an assigned UPC, would be associated with fewer asthma-related ED visits and hospitalizations.

## Methods

### Study design and setting

We conducted a population-based retrospective cohort study with linked administrative data across outpatient and inpatient health settings from the province of Quebec, Canada, for children aged 2-16 years old, with a diagnosis of asthma from January 1, 2010, to December 31, 2011.

### Data sources and characteristics of participants

Québec is Canada’s second-largest province in terms of population, with approximately 8.2 million inhabitants [11]. All Québec permanent residents have access to public health insurance, administered by the Régie de l’Assurance Maladie du Québec (RAMQ), covering all essential medical services provided in hospitals or outpatient settings. We used three databases, linked together using an encrypted health number [12]: 1) the Registered Persons Database (encrypted health insurance number, sex, age, and postal code); 2) the Physician Claims Database (records of remunerated services through all clinical settings, i.e., RAMQ billings); and 3) the Hospital Discharge Database (MED-ECHO, all admissions data from the hospitals). Rurality and socioeconomic status were assigned by linking postal codes from the registered person’s databases to 2011 Statistics Canada census data.

We used a validated algorithm to identify those children with administratively defined asthma as of December 31, 2011. This definition required at least two physician visits or one hospitalization for asthma in the RAMQ billings during the exposure period of January 1, 2010, and December 31, 2011 [13, 14]. We excluded patients with invalid health insurance numbers.

### Primary exposure: usual provider of primary care

We assigned each child to one of the four types of UPC: family physicians within the team-based FMG, family physicians not part of an FMG, pediatrician, or no assigned UPC using the RAMQ physician claims during two-year exposure of January 1, 2010, to December 31, 2011. To assign the UPC, we adapted an algorithm created to identify patient attachment to a family physician amongst adults with RAMQ data, which we have previously used in the pediatric population (see Appendix I) [15, 16]. The algorithm used a hierarchy, in which we first searched for billing codes identifying that a patient was enrolled with a team-based FMG or family physician not part of an FMG, or followed for routine growth and development monitoring by a pediatrician. If these codes were not available, the patient was assigned to the usual provider of care who billed the most primary care visits (with a minimum of 2 visits). The remainder of patients who did not satisfy the aforementioned criteria had no UPC.

### Secondary exposure: usual provider of care (UPC) index score

Continuity of care between patients and providers has been previously formulated and categories into the following: interpersonal continuity (the ongoing personal relationship between patient and physician), longitudinal continuity (the accumulation of interactions over a period of time), informational continuity (the availability and exchange of medical and social information over time and between professionals), and management continuity (the effective execution of a care plan through collaboration and coordination of health care teams) [17, 18].

We examined longitudinal continuity in the current study through the use of the UPC Index. The UPC Index was defined as the proportion of a child’s medical visits with their assigned UPC [5]. This measure takes on a value of 0 to 1, with values close to 1 suggesting a high continuity of care. The UPC Index was divided into 3 categories a priori (>0-0.4= low, 0.41-0.70 = medium, >0.70 = high) as in previous studies [17–19]. The score was assigned to each child by dividing the total number of visits with the child’s determined UPC (n_i_) by the total number of primary care visits with any primary care provider (n) between January 1, 2010, and December 31, 2011, (Eq. 1) [20].

$$UPC\ Index=\max \frac{n_i}{n}$$

## Equation 1 usual provider of care (UPC) index [20]

### Outcomes

The primary and secondary outcomes were asthma-related ED visits and hospitalizations, respectively, measured in the two-year outcome follow-up period of January 1, 2012, to December 31, 2013, as binary outcomes. ED visits were determined through the identification of physician claims where the establishment code was the ED. Hospital admissions were determined using the MED-ECHO database. Outcomes were determined to be “asthma-related” by using ICD-9 (for ED visits) and ICD-10 (for hospitalization) codes agreed upon by Québec asthma specialists (Appendix 1), identified in the Physician Claims Database and the MED-ECHO databases, respectively [21].

### Covariates

The covariates were age, sex, socioeconomic status (SES), rurality, other co-morbidities, and previous health care utilization. Children were categorized into the following age groups: 2-5 years old, 6-9 years old, 10-12 years old, and 13-16 years old. SES was determined using the Material and Social Deprivation Index, which is based on census data [22]. The study population was divided into five quintiles (Q1 to Q5, least deprived to most deprived). Rurality was defined using the Census Metropolitan and Census Agglomeration Influenced Zone developed by Statistics Canada and divided into 3 categories: urban (population>100,000), small cities (population 10,000- 100,000), and rural (population <10,000) [23]. To account for other co-morbidities, specifically prevalent chronic diseases associated with higher healthcare utilization (i.e., diabetes and children with medical complexity), children were classified as having asthma only or asthma and other chronic diseases [24]. Previous health care utilization was measured by previous all-cause ED visits, all-cause hospital admissions, and asthma specialist (either a pediatrician who billed for an asthma visit in a hospital outpatient clinic and/or a pulmonologist) visits between 2010-2011.

### Statistical analysis

Medians and interquartile ranges (IQR) and the counts and percentages were reported to summarize the distribution of continuous and categorical variables, respectively.

To test the association between the exposures and the outcomes, multivariable logistic regression models were used, and results were reported as odds ratios (OR) with 95% confidence intervals (CI). The models were adjusted with all the covariates described in the preceding section. Given that we anticipated <5% of missing data based on previous work with similar Quebec health administrative data, we planned to exclude missing values from the analyses [16]. All statistical analyses were completed in SAS software, Version 9.4 (SAS Institute, Inc., Cary, NC, USA).

### Sensitivity analyses

To assess the robustness of our findings with the secondary exposure (UPC Index), we conducted a sensitivity analysis using a different measure of continuity of care, the Bice-Boxerman (COC) Index. The COC index measures the dispersion of care (numerator in Eq. 2) over one or several primary care providers (denominator in Eq. 2) [25]. This measure takes on a value of 0 to 1, with values close to 1, suggesting a high continuity of care. We constructed the COC Index using only primary care visits and the following Eq. 2 [25].

$$COC\ Index=\frac{\left(\sum_{i=1}^p{n}_i^2\right)-n}{n\left(n-1\right)}$$

(where n is the total number of primary care visits, *n*_*i*_ is the number of visits with primary care physician *i*, and *p* is the total number of primary care physicians visited [25])

## Equation 2 bice-boxerman continuity of care (COC) Index [25]

### Ethics approval

In Quebec, the use of health administrative data for research projects is highly regulated and monitored, and must be approved by the Commission d’accès à l’information and a research ethics board. The health administrative data is anonymized, and extensive measures are in place to ensure confidentiality and ethical conduct of research. Thus, informed consent was not required. All methods were carried out in accordance with relevant guidelines and regulations. In the current study, we obtained the required approval by the Commission d’accès à l’information and the REB at the McGill University Health Centre.

## Results

We identified 39,341 children with administratively defined asthma (Table 1). As of January 1, 2012, 17.4% of children diagnosed with asthma had no assigned UPC. The majority of the patient population was followed by a pediatrician (34.9%), then by team-based FMG (24.1%), and finally by non-FMG (23.6%). The median [IQR] number of visits made to the UPC were 4 [3, 7], 3 [2, 4], and 3 [1, 5] for pediatrician, team-based FMG, and non-FMG, respectively. Children who were determined to have no UPC, in comparison to other primary care models, were more likely to come from the older age categories, come from the most deprived socioeconomic quintile, and live in non-urban settings.

**Table 1:** Baseline characteristics of cohort by primary care model

Variable	Type of primary care model				All N (%)
	Pediatrician N (%)	FMG N (%)	Non-FMG N (%)	No assigned UPC N (%)	
**Total**	13,743 (34.9)	9,464 (24.1)	9,286 (23.6)	6,848 (17.4)	39,341 (100)
**Age category**					
2-5 years old	6,346 (46.2)	3,958 (41.8)	4,000 (43.1)	1,822 (26.6)	16,126 (41.0)
6-9 years old	4,032 (29.3)	2,587 (27.3)	2,353 (25.3)	2,065 (30.2)	11,037 (28.1)
10-12 years old	1,937 (14.1)	1,298 (13.7)	1,347 (14.5)	1,415 (20.7)	5,997 (15.2)
13-16 years old	1,428 (10.4)	1,621 (17.1)	1,586 (17.1)	1,546 (22.6)	6,181 (15.7)
**Sex**					
Female	5,402 (39.3)	3,918 (41.4)	3,758 (40.5)	2,693 (39.3)	15,771 (40.1)
**SES**					
Q1 (least deprived)	3,951 (28.8)	2,134 (22.6)	1,827 (19.7)	1,358 (19.8)	9,270 (23.6)
Q2	2,984 (21.7)	2,415 (25.5)	1,996 (21.5)	1,346 (19.7)	8,741 (22.2)
Q3	2,211 (16.1)	1,846 (19.5)	1,713 (18.5)	1,209 (17.7)	6,979 (17.7)
Q4	2,112 (15.4)	1,464 (15.5)	1,552 (16.7)	1,236 (18.1)	6,364 (16.2)
Q5 (most deprived)	2,053 (14.9)	1,272 (13.4)	1,839 (19.8)	1,385 (20.2)	6,549 (16.7)
Missing	432 (3.1)	333 (3.5)	359 (3.9)	314 (4.6)	1,438 (3.7)
**Rurality**					
Urban (population >100k)	11,821 (86.0)	5,473 (57.8)	6,658 (71.7)	4,574 (66.8)	28,526 (72.5)
Small cities (population 10k-100k)	823 (6.0)	1,702 (18.0)	967 (10.4)	955 (14.0)	4,447 (11.3)
Rural (Population <10k)	1,004 (7.3)	2,228 (23.5)	1,595 (17.2)	1,262 (18.4)	6,089 (15.5)
Missing	95 (0.7)	61 (0.6)	66 (0.7)	57 (0.8)	279 (0.7)
**Other co-morbidities**					
Asthma only	12,536 (91.2)	8,688 (91.8)	8,460 (91.1)	6,004 (87.7)	35,688 (90.7)
Asthma and other chronic diseases	1,207 (8.8)	776 (8.2)	826 (8.9)	844 (12.3)	3,653 (9.3)
**Previous ed visits**					
0 Visits	6,016 (43.8)	3,392 (35.8)	3,270 (35.2)	2,326 (34.0)	15,004 (38.1)
1 Visit	2,857 (20.8)	1,850 (19.6)	1,844 (19.9)	1,513 (22.1)	8,064 (20.5)
2-3 Visits	2,743 (20.0)	2,130 (22.5)	2,137 (23.0)	1,676 (24.5)	8,686 (22.1)
>4 Visits	2,127 (15.5)	2,092 (22.1)	2,035 (21.9)	1,333 (19.5)	7,587 (19.3)
**Previous hospital admissions**					
Yes	3,166 (23.0)	3,233 (34.2)	3,307 (35.6)	2,232 (32.6)	11,938 (30.3)
**Previous asthma specialist visits**					
0 visits	902 (6.6)	5,058 (53.4)	4,649 (50.1)	1,867 (27.3)	12,476 (31.7)
1 visits	1,672 (12.2)	1,445 (15.3)	1,499 (16.1)	1,125 (16.4)	5,741 (14.6)
2 visits	5,601 (40.8)	1,389 (14.7)	1,403 (15.1)	2,003 (29.2)	10,396 (26.4)
>3 visits	5,568 (40.5)	1,572 (16.6)	1,735 (18.7)	1,853 (27.1)	10,728 (27.3)

*Legend*: *ED* Emergency Department, *SES* Socioeconomic status, *Q* Socioeconomic Quintile, *FMG* Family Medicine Groups, *UPC* Usual Provider of Care

Table 2 shows the crude proportions and adjusted odd ratios of asthma-related ED visits and hospital admissions for the main exposure, UPC, and the covariates. A total of 10.3% and 6.1% of the cohort had asthma-related ED visits and hospital admissions, respectively. Children who had no UPC had the highest percentage of experiencing asthma-related ED visits (12.1%) and hospital admissions (8.2%). We found that overall, children who had any type of primary care physician compared to those without, had a decreased odds of having asthma-related ED visit (pediatrician OR: 0.80, 95% CI [0.73, 0.88]; team-based FMG OR: 0.84, 95% CI [0.75,0.93]; non-FMG OR: 0.92, 95% CI [0.83, 1.02]) or hospital admission (pediatrician OR: 0.66, 95% CI [0.58, 0.75]; team-based FMG OR: 0.82, 95% CI [0.72, 0.93]; non-FMG OR: 0.76, 95% CI [0.67, 0.87]).

**Table 2:** Curde proportions and adjusted odds ratios of asthma-related acute outcomes for the main exposure, UPC, and covariates

Variable	No. (%) of children with an ED visit, by variable	ED visits or not, OR (95% CI)	No. (%) of children with a hospital admission, by variable	Hospital admission or not, OR (95% CI)
**Primary care model**				
No Assigned UPC	826/6,848 (12.1)	Reference	558/6,848 (8.2)	Reference
Pediatrician	1,225/13,743 (8.9)	0.80 (0.72, 0.88)	643/13,743 (4.7)	0.66 (0.58, 0.75)
FMGs	961/9,464 (10.2)	0.84 (0.75, 0.93)	618/9,464 (6.5)	0.82 (0.72, 0.93)
Non-FMGs	1,054/9,286 (11.4)	0.92 (0.83, 1.02)	586/9,286 (6.3)	0.76 (0.67, 0.87)
**Age category**				
2-5 yo	2,060/16,126 (12.8)	Reference	1,241/16,126 (7.7)	Reference
6-9yo	1,035/ 11,037 (9.4)	1.09 (1.00, 1.18)	552/11,037 (5.0)	0.91 (0.81, 1.02)
10-12yo	518/5,997 (8.6)	1.17 (1.05, 1.30)	281/5,997 (4.7)	0.89 (0.77, 1.03)
13-16yo	453/6181 (7.3)	0.94 (0.84, 1.06)	331/6,181 (5.4)	0.90 (0.79, 1.04)
**Sex**				
Female	1,487/15,771 (9.4)	Reference	964/15,771 (6.1)	Reference
Male	2,579/23,570 (10.9)	1.08 (1.01, 1.16)	1,442/23,570 (6.1)	0.95 (0.87, 1.04)
**SES**				
Q1 (least deprived)	732/9,270 (7.9)	Reference	473/9,270 (5.1)	Reference
Q2	829/8,741 (9.5)	1.07 (0.96, 1.18)	513/8,741 (5.9)	1.02 (0.89, 1.16)
Q3	728/6,979 (10.4)	1.10 (0.99, 1.23)	469/6,979 (6.7)	1.10 (0.97, 1.27)
Q4	749/6,364 (11.8)	1.24 (1.11, 1.38)	397/6,364 (6.2)	1.03 (0.90, 1.19)
Q5 (most deprived)	862/6,549 (13.2)	1.34 (1.21, 1.49)	469/6,549 (7.2)	1.12 (0.98, 1.29)
**Rurality**				
Urban (population >100k)	2,771/28,526 (9.7)	Reference	1,551/28,526 (5.4)	Reference
Small cities (population 10k-100k)	498/4,447 (11.2)	0.97 (0.87, 1.08)	362/4,447 (8.1)	1.25 (1.10, 1.43)
Rural (population <10k)	781/6,089 (12.8)	1.07 (0.98, 1.18)	482/6,089 (7.9)	1.12 (1.00, 1.27)
**Other co-morbidities**				
Asthma Only	3,619/35,688 (10.1)	Reference	1,539/35,688 (4.3)	Reference
Asthma & other comorbidities	447/3,653 (12.2)	0.81 (0.73, 0.91)	866/3,653 (23.7)	4.68 (4.24, 5.17)
**Previous hospital admission**				
No	2,163/27,403 (7.9)	Reference	910/27,403 (3.3)	Reference
Yes	1,903/11,938 (15.9)	1.09 (1.01, 1.17)	1,495/11,938 (12.5)	1.95 (1.76, 2.15)
**Previous Ed visits**				
0 Visit	442/15,004 (2.9)	Reference	380/15,004 (2.5)	Reference
1 Visit	586/8,064 (7.3)	2.47 (2.17, 2.81)	364/8,064 (4.5)	1.41 (1.21, 1.64)
2-3 Visits	1,101/8,686 (12.7)	4.47 (3.98, 5.03)	599/8,686 (6.9)	1.92 (1.67, 2.21)
Over 4 visits	1,937/7,587 (25.5)	10.13 (9.00, 11.39)	1,062/7,587 (14.0)	3.12 (2.72, 3.59)
**Previous asthma specialist visits**				
0 Visits	1,068/12,476 (8.6)	Reference	739/12,476 (5.9)	Reference
1 Visit	624/5,741 (10.9)	1.15 (1.03, 1.29)	330/5,741 (5.8)	0.94 (0.81, 1.08)
2 Visits	814/10,396 (7.8)	0.99 (0.89, 1.10)	385/10,396 (3.7)	0.82 (0.72, 0.95)
Over 3 Visits	1,560/10,728 (14.5)	1.51 (1.37, 1.66)	951/10,728 (8.9)	1.35 (1.20, 1.51)

Logistic model adjusted for all covariates in the table

*Legend*: *ED* Emergency Department, *SES* Socioeconomic Status, *Q* Socioeconomic Quintile, *FMG* Family Medicine Groups, *UPC* Usual Provider of Care

Among children who had a UPC (82.6%), 37.4% had a low UPC Index score (Table 3). Children who had low continuity of care had a median of 2 (IQR: [2, 3]) visits with their UPC and 10 (IQR: [6, 14]) primary care visits in total over the two-year exposure period. In contrast, those children who had high continuity of care had a median of 5 (IQR: [3, 8]) visits with their UPC and a median of 6 (IQR: [4, 10]) primary care visits in total. We found that those children who had high continuity of care were more likely to be followed by a pediatrician (59.4%) than those who had low continuity who were more likely to be followed by a family physician in a team-based FMG (40.5%). Those children who had high continuity of care with their UPC, in comparison to low, were more likely to come from the most affluent neighborhood, come from an urban setting, or have no prior ED visits and hospital admissions.

**Table 3:** Baseline characteristics of cohort by UPC index

Variables	Level of continuity of care by UPC index			ALL N (%)
	Low continuity of care (>0-0.40) N (%)	Medium continuity of care (>0.40-0.70) N (%)	High continuity of care ( >0.70) N (%)	
**Total**	12,148 (37.4)	10,702 (32.9)	9,643 (29.7)	32,493 (100.0)
**Primary care model**				
Pediatrician	3,028 (24.9)	4,992 (46.6)	5,723 (59.4)	13,743 (42.3)
FMG	4,920 (40.5)	2,791 (26.1)	1,753 (18.2)	9,464 (29.1)
Non-FMG	4,200 (34.6)	2,919 (27.3)	2,167 (22.5)	9,286 (28.6)
**Age category**				
2-5 years old	6,411 (52.8)	4,572 (42.7)	3,321 (34.4)	14,304 (44.0)
6-9 years old	3,236 (26.6)	2,975 (27.8)	2,761 (28.6)	8972 (27.61)
10-12 years old	1,275 (10.5)	1,568 (14.6)	1,739 (18.0)	4,582 (14.1)
13-16 years old	1,226 (10.1)	1,587 (14.8)	1,822 (18.9)	4,635 (14.3)
**Sex**				
Female	4,815 (39.6)	4,312 (40.3)	3,951 (41.0)	13,078 (40.3)
**SES**				
Q1 (least deprived)	2,715 (22.4)	2,583 (24.1)	2,614 (27.1)	7,912 (24.4)
Q2	2,871 (23.6)	2,461 (23.0)	2,063 (21.4)	7,395 (22.8)
Q3	2,213 (18.2)	1,943 (18.2)	1,614 (16.7)	5,570 (17.8)
Q4	1,945 (16.0)	1,701 (15.9)	1,482 (15.4)	5,128 (15.8)
Q5 (most deprived)	1,934 (15.9)	1,660 (15.5)	1,570 (16.3)	5,164 (15.9)
Missing	470 (3.9)	354 (3.3)	300 (3.1)	1,124 (3.5)
**Rurality**				
Urban (population >100k)	8,182 (67.4)	8,084 (75.5)	7,686 (79.7)	23,952 (73.7)
Small cities (population 10k-100k)	1,655 (13.6)	1,024 (9.6)	813 (8.4)	3,492 (10.8)
Rural (population <10k)	2,229 (18.4)	1,523 (14.2)	1,075 (11.2)	4,827 (14.9)
Missing	82 (0.7)	71 (0.7)	69 (0.7)	222 (0.7)
**Other co-morbidities**				
Asthma only	10,975 (90.3)	9,804 (91.6)	8,905 (92.4)	29,684 (91.4)
Asthma and other chronic diseases	1,173 (9.7)	898 (8.4)	738 (7.6)	2,809 (8.6)
**Previous Ed visits**				
0 Visit	3,085 (25.4)	4,017 (37.5)	5,576 (57.8)	12,678 (39.0)
1 Visit	2,175 (17.9)	2,417 (22.6)	1,959 (20.3)	6,551 (20.2)
2-3 Visits	3,077 (25.3)	2,534 (23.7)	1,399 (14.5)	7,010 (21.6)
Over 4 Visits	3,811 (31.4)	1734 (16.20)	709 (7.35)	6254 (19.25)
**Previous hospital admission**				
Yes	5,157 (42.5)	2,989 (27.9)	1,560 (16.2)	9,706 (29.9)
**Previous asthma specialist visits**				
0 Visit	4,421 (36.4)	3,472 (32.4)	2,716 (28.2)	10,609 (32.7)
1 Visit	2,139 (17.6)	1,560 (14.6)	917 (9.5)	4616 (14.21)
2 Visits	2,560 (21.1)	2,764 (25.8)	3,069 (31.8)	8393 (25.83)
Over 3 Visits	3,028 (24.9)	2,906 (27.2)	2,941 (30.5)	8875 (27.31)

*Legend*: *ED* Emergency Department, *SES* Socioeconomic status, *Q* Socioeconomic Quintile, *FMG* Family Medicine Groups, *UPC* Usual Provider of Care

Table 4 shows the crude proportions and adjusted ORs of asthma-related ED visits and hospital admissions for the secondary exposure (the UPC Index) among children who had a UPC. The low UPC Index group had the highest percentage of children who experienced an asthma-related ED visit (12.9%) and hospital admission (7.8%). There were no significant differences in the adjusted analyses for ED visits. Compared to low continuity, both medium and high continuity of care was associated with decreased odds of hospitalizations, but the associations were not statistically significant.

**Table 4:** Crude proportions and adjusted odds ratios of asthma-related acute outcomes for the secondary exposure, UPC Index, and covariates

Variables	No. (%) of children with an ED visit, by variable	ED visits or not, OR (95% CI)	No. (%) of children with a hospital admission, by variable	Hospital admission or not, OR (95% CI)
**UPC index**				
Low (>0-0.40)	1,566/12,148 (12.9)	Reference	942/12,148 (7.8)	Reference
Medium (>0.40-0.70)	1,002/10,702 (9.4)	0.98 (0.90, 1.08)	559/10,702 (5.2)	0.93 (0.82, 1.04)
High (>0.70)	672/9,643 (7.0)	1.06 (0.95, 1.19)	346/9,643 (3.6)	0.87 (0.75, 1.01)
**Primary care model**				
FMG	961/9,464 (10.1)	Reference	618/9,464 (6.5)	Reference
Pediatrician	1,225/13,743 (8.9)	0.96 (0.86, 1.07)	643/13,743 (4.7)	0.84 (0.73, 0.97)
Non-FMGs	1,054/9,286 (11.3)	1.11 (1.00, 1.22)	586/9,286 (6.3)	0.94 (0.83, 1.06)
**Age category**				
2-5 yo	1,765/14,304 (12.3)	Reference	1,041/14,304 (7.3)	Reference
6-9yo	789/8,972 (8.8)	1.10 (1.00, 1.21)	407/8,972 (4.5)	0.95 (0.83, 1.07)
10-12yo	376/4,582 (8.2)	1.22 (1.08, 1.39)	192/4,582 (4.2)	0.95 (0.80, 1.12)
13-16yo	310/4,635 (6.7)	0.95 (0.83, 1.09)	207/4,635 (4.5)	0.93 (0.79, 1.10)
**Sex**				
Female	1,153/13,078 (8.8)	Reference	724/13,078 (5.5)	Reference
Male	2,087/19,415 (10.7)	1.13 (1.04, 1.22)	1,123/19,415 (5.8)	0.97 (0.88, 1.07)
**SES**				
Q1 (least deprived)	613/7,912 (7.8)	Reference	382/7,912 (4.8)	Reference
Q2	688/7,395 (9.3)	1.07 (0.955, 1.20)	422/7,395 (5.7)	1.04 (0.90, 1.20)
Q3	585/5,770 (10.1)	1.11 (0.99, 1.26)	361/5,770 (6.3)	1.11 (0.95, 1.29)
Q4	597/5,128 (11.6)	1.28 (1.13, 1.44)	300/5,128 (5.8)	1.05 (0.89, 1.23)
Q5 (most deprived)	638/5,164 (12.4)	1.31 (1.16, 1.47)	322/5,164 (6.2)	1.08 (0.93, 1.27)
**Rurality**				
Urban (population >100k)	2,230/23,952 (9.3)	Reference	1,190/23,952 (5.0)	Reference
Small cities (population 10k-100k)	391/3,492 (11.2)	0.99 (0.88, 1.12)	290/3,492 (8.3)	1.35 (1.16, 1.56)
Rural (population <10k)	610/4,827 (12.6)	1.08 (0.97, 1.20)	360/4,827 (7.5)	1.14 (1.00, 1.31)
**Other co-morbidities**				
Asthma Only	2,901/29,684 (9.8)	Reference	1,269/29,684 (4.3)	Reference
Asthma & other comorbidities	339/2,809 (12.1)	0.82 (0.72, 0.94)	578/2,809 (20.6)	3.95 (3.52, 4.44)
**Previous hospital admission**				
No	1,707/22,787 (7.5)	Reference	710/22,787 (3.1)	Reference
Yes	1,533/9,706 (15.8)	1.08 (0.99, 1.17)	1,137/9,706 (11.7)	1.91 (1.70, 2.14)
**Previous ed visits**				
0 Visit	353/12,678 (2.8)	Reference	278/12,678 (2.2)	Reference
1 Visit	448/6,551 (6.8)	2.50 (2.16, 2.88)	256/6,551 (3.9)	1.41 (1.18, 1.69)
2-3 Visits	857/7,010 (12.2)	4.67 (4.08, 5.33)	474/7,010 (6.8)	2.13 (1.81, 2.50)
Over 4 visits	1,582/6,254 (25.3)	10.86 (9.48, 12.43)	839/6,254 (13.4)	3.34 (2.83, 3.93)
**Previous asthma specialist visitS**				
0 Visits	880/10,609 (8.3)	Reference	551/10,609 (5.2)	Reference
1 Visit	497/4,616 (10.8)	1.16 (1.02, 1.31)	261/4,616 (5.6)	1.02 (0.87, 1.20)
2 Visits	611/8,393 (7.3)	0.97 (0.86, 1.09)	295/8,393 (3.5)	0.89 (0.76, 1.05)
Over 3 Visits	1,252/8,875 (14.1)	1.49 (1.34, 1.66)	740/8,875 (8.3)	1.45 (1.27, 1.65)

Logistic model adjusted for all covariates in the table

*Legend*: *ED* Emergency Department, *SES* Socioeconomic status, *Q* Socioeconomic Quintile, *FMG* Family Medicine Groups, *UPC* Usual Provider of Care

### Sensitivity analyses

In the sensitivity analyses using the COC Index, the results were similar when using the UPC Index, but some associations were significant (see Additional files: Appendix 2). Compared to those who had a low COC Index score, children who had a high COC Index score had an increased odds of having an asthma-related ED visit (high OR: 1.10, 95% CI [1.01, 1.21]). Children who had a medium COC Index score had a decreased odds of having an asthma-related hospital admission compared to those who had a low COC Index score (medium OR: 0.84, 95% CI [0.72, 0.98]).

## Discussion

Using a population-based cohort of children with asthma in Quebec (*N* = 39,341), we demonstrated that 17.4% did not have an assigned UPC, and for those who had an assigned UPC, 38.1% had low continuity of care. Having a UPC compared to having no assigned UPC was associated with reduced asthma-related ED visits and hospital admissions. Children with the lowest continuity of care (UPC Index) compared to medium or high continuity of care experienced higher rates of asthma-related ED visit (12.9% vs. 9.4% or 7.0%, respectively) and hospital admission (7.8% vs. 5.2 or 3.6%, respectively). However, in the adjusted analyses, the associations between continuity of care and outcomes were not significant.

Our findings are in line with several studies conducted in the general adult population. These studies have shown that having a regular source of care compared to none was associated with decreased odds of an ED visit [7, 26–31]. In a telephone survey of 8 502 Ontario residents 16 years and older, among those with a chronic disease, having a regular family physician was associated with a decreased likelihood of ED use (OR=0.47, *p* = 0.01) [32]. Glazier et al. [33] also found that patients from the general population with at least one chronic condition and without a family physician were 1.22 times more likely to have an ED visit than those who had a regular physician. A study of Medicaid-insured children in the United States also demonstrated that increased preventive asthma visits and acute asthma care by primary care pediatricians was associated with decreased ED visits and hospitalizations, supporting the role of regular assessment and monitoring by primary care [34, 35]. In the current study, the increased ED visits by children without a UPC may have been explained by the use of the ED by these children for drug renewals or treatment of minor asthma exacerbations that could otherwise have been managed in primary care [36, 37].

In the Canadian setting where access to health care is universal, we observed socioeconomic inequalities. Compared to other primary care models, children with no UPC were more likely to come from the most deprived socioeconomic quintile. Further, children from the most compared to the least deprived quintile were more likely to have ED visits (OR: 1.34, 95% CI [1.21, 1.49]) and hospitalizations (OR: 1.12, 95% CI [0.98, 1.29]). A recent scoping review mapped out the multiple structural and social determinants of health related to asthma that are associated with poor outcomes, such as access to healthcare, medications, education, and housing [38]. Reducing disparities in asthma outcomes requires interventions that can, at least in part, effectively address these interconnected determinants. For example, previous studies have evaluated community health workers who provided psychosocial and educational support, care coordination, home environment assessment, and remediation. These interventions were reported to be cost-effective, as well as reduce ED visits, hospitalizations, patient missed school days, and parent missed workdays [39–41].

Children whose assigned UPC was a pediatrician, compared to other models, had decreased odds of having asthma-related ED visits and hospital admissions. Possible explanations for these findings include increased availability of walk-in clinics to prevent ED visits or better adherence to evidence-based treatments to prevent exacerbations amongst pediatricians. However, for the latter hypothesis, a survey conducted in Quebec around the same time as the current study (2013-2014) found that pediatricians and family physicians did not differ in their approach to prescribing long-term controller medication for patients with persistent asthma [42]. In the current study, children assigned to a pediatrician were also more likely to have high continuity of care. Clinic-related factors have been shown to predict higher continuity of care (as reported by patients) in primary care practices in Ontario, Canada [43], including having more than 24 hours on call per week for physicians, having a smaller practice, having fewer nurses, and being closed on weekends (so patients could not see whichever family physician was covering the clinic on the weekend, thus decreasing continuity with their primary provider) [43].

Although the associations were not significant, high continuity of care with a UPC was associated with increased odds of having an ED visit and decreased odds of having a hospital admission. Prior studies examining these associations have produced mixed results and had some limitations, which the current study attempted to address [6, 7, 19, 27, 44]. These limitations included a focus on a specific population (such as Medicaid recipients or US-based private medical insurance cooperative) [6, 7, 27, 44], a cross-sectional design [27], or a lack of pediatric focus [19, 27]. Cree et al. [19], which was the only study conducted in Canada using administrative data from 2774 children and adults with asthma limited to one health region in Alberta, found that high continuity of care was associated with decreased risk of an ED visit (OR= 0.24, 95% CI [0.19-0.29]) and a decreased risk of the number of hospitalizations (RR=0.69, 95% CI [0.54-0.89]). In the current study, the increased odds of ED visits among those with high continuity of care may signal issues around timely access to the UPC during an asthma exacerbation. Hospitalizations generally represent a more severe asthma exacerbation. Higher continuity of care with a UPC may have played a role in better controlling the disease to prevent a more severe asthma presentation.

Our study had some limitations. Firstly, although we adjusted for multiple variables, there may have been residual confounders not captured in our population-based health administrative database, such as adherence to prescribed medication, asthma phenotype (i.e. specific clinical, biological, physiological characteristics), and physician characteristics. Secondly, we adjusted for previous ED visits as a proxy for clinical factors that we cannot measure in the health administrative data, such as children who have more severe asthma phenotypes that required ED visits. However, we may have overadjusted our regression models by including previous ED visits as a potential confounder. The latter would have occurred if a given UPC group, being a model with less accessible primary care, resulted in ED visits, both prior to *and* during the outcome assessment periods. In this instance, adjusting for previous ED visits could have absorbed some of the effect of the UPC exposure; instead of the effect being attributed to the UPC exposure some of it would be attributed to prior ED visits. Therefore, differences between the UPC groups may be more pronounced than reported by our findings. Thirdly, although the UPC Index and the COC Index are among the most used administrative measures of continuity in primary care research, the UPC index, only captures one aspect of continuity of care, longitudinal continuity. It does not consider other domains of continuity of care, such as management or interpersonal continuity [5]. We attempted to address the former through our sensitivity analysis using the COC Index, which attempts to measure management continuity, i.e., the effective collaboration and coordination of health care teams [20]. Some studies have demonstrated an association between interpersonal continuity and improved preventive care and reduced hospitalizations [45]. The UPC Index used in our analyses measures longitudinal continuity and is not a direct measure of interpersonal continuity, although concepts may overlap; repeated interactions (longitudinal continuity) may lead to a therapeutic relationship (interpersonal continuity), but it is not guaranteed that seeing the same doctor equates to a good patient-doctor relationship [46], or to better outcomes. Lastly, no matter the quality of primary care services received, especially in young populations, some acute care utilization is unavoidable, and administrative data did not allow us to differentiate these visits from those that could be avoided by timely and effective primary care.

## Conclusions

In a universal health care system, the current study revealed the importance of access, and potentially continuity of care, with a usual provider of care for reducing asthma-related ED visits and hospital admissions.

## Supplementary Information

**Additional file 1.**

## Data Availability

The datasets generated during the current study are not publicly available due privacy laws by the Commission d’accès à l’information of Quebec but analyzed data may be available from the corresponding author on reasonable request.

## Abbreviations

ED: Emergency Department
FMG: Family Medicine Groups
UPC: Usual Provider of Care
RAMQ: Régie de l’Assurance Maladie du Québec
COC: Bice-Boxerman Continuity of Care Index
OR: Odds Ratio
CI: Confidence Interval
CMC: Children with Medical Complexity
Q: Socioeconomic Quintile
IQR: Interquartile Range
