# Supplementary material for: Are primary care and continuity of care associated with asthma-related acute outcomes amongst children? A retrospective population-based study
Source: BMC Prim Care. 2022 Jan 14;23:5. doi: 10.1186/s12875-021-01605-7 (PMC8759282; doi:10.1186/s12875-021-01605-7)
Supplement: Supplementary file 1 — Additional file 1. [file 12875_2021_1605_MOESM1_ESM.docx]

## APPENDIX 1: DETAILED METHODS

### Primary Exposure

The primary exposure of interest for the first objective of this project was the primary care model. Children with asthma were assigned to one of the four possible primary care models; family physicians in FMGs, family physicians in non-FMGs, pediatricians or no primary care. This was determined by assigning a “usual provider of care” by looking at RAMQ data and visits from January 1, 2010 to December 31, 2011. The usual provider of care for each child was determined according to the algorithm below in Table 5.

**Table 5 Algorithm for determining usual provider of care (UPC)**

| **Algorithm to identify “usual provider of care (UPC)”** |
| --- |
| **STEP 1**  Use codes for “enrollment” under a family physician. If subject has one of the following codes, then “primary care model” is a family doctor (FMG or non-FMG): 08875, 08877, 15144, 15145, 00059, 15158, 15159, 15148, 15169, 15170, 15171, 19952, 19951, 19954, 19955, 15156, 15157, 15189, 19074  The “Usual Provider of Care” is the family physician who billed any of the above codes, except for 19074 |
| **STEP 2**  If subjects do not have a code identifying a family physician, search for enrollment by a pediatrician using the 09194 code. This code is not specific to “enrollment” of patients under a pediatrician but it is used by pediatricians for follow-up or growth and development milestones. If this code is found, the “primary care model” is pediatrician.  The “usual provider of care” is the pediatrician who has billed the most 09194 codes. |
| **STEP 3**  If a subject does not have a code identifying a family physician or pediatrician, calculate the number of visits by a family physician (09092, 08870 (00005), 08871 (00056), 08872 (00097), 08901 (08807), 08902 (08809), 15161, 15230, 00474, 00002, 08873, 08874, 08855, 00007, 00075—brackets indicate these codes are billed by CHSGS/CLSC* outpatient clinic—and for each visits by a pediatrician (09129, 09127, 09171, 09172 – ALL billed by a pediatrician and not any other specialist).  Only one act per day per doctor can be included when calculating number of visits. Only physicians with at least 2 visits can be considered for STEP 3. The following are ways that a usual provider of care can be assigned in STEP 3:  a. Family Physician (FMG or non-FMG) is assigned for the “primary care model”: if the number of visits by the **same** family physician > the number of visits by the **same** pediatrician. The “usual provider of care” in this case is the family physician with the most complete major exams (00872 or 00097). If there are no complete major exams, select the family physician with the most visits.  b.Pediatrician is assigned for the "primary care model”: if the number of visits by the **same** pediatrician > the number of visits by the **same** family physician. The “usual provider of care” is the pediatrician with the most visits.  c. For the “primary care model” if the number of visits (>=2) by the **same** pediatrician = number of visits (>=2) by the **same** family physician, then Family Physician (FMG or non-FMG) is assigned if there are at least 2 complete major exams (00872, 00097) by the same family physician; otherwise, Pediatrician is assigned. For the “usual provider of care”, if Family physician is assigned as the “primary care model”, select the family physician with the most complete major exams (00872 or 00097). If there are no complete major exams, select the family physician with the most visits. If the “primary care model” is Pediatrician, the “usual provider of care” is the pediatrician with the most visits. |
| **STEP 4**  If no UPC is identified through STEPS 1 through 3, then the subject does not have a UPC and is classified as “no primary care”. |

If the usual provider of care was determined to be a family physician, Table 6 was used to determine if the model of care was a FMG or a non-FMG. The codes were displayed below are in a hierarchy, therefore billing code 08875 was searched for first, followed by billing code19074, then looking at the establishment code of each FMG.

**Table 6 Algorithm to determine family physicians in FMGs**

| **Code** | **Coding** | **Details** |
| --- | --- | --- |
| First, use the billing code  08875 (for any visits) | FMG | Inscription of patients in FMG |
| Then, use billing code 19074 (for any visits) | FMG | Temporary inscription of pregnant patient in FMG (followed by another MD in the same FMG) |
| Then look at list of FMGs. If patient had visit with UPC (family physician) at any time from Jan 1, 2010-December 31, 2011 in an establishment listed as an FMG during Jan 1 2010 to December 31, 2011, then primary care model is FMG. | FMG | Medical clinic coded for Family Medicine Groups |

### Secondary Exposure

The secondary exposure variable for the secondary objective was the Usual Provider of Care Index. A child’s usual provider of care (UPC) Index score was determining by looking at visit with their usual provider of care determined in Table 5 between January 1,2010 and December 31, 2011. The algorithm presented in Table 7 was used to determine the UPC Index score each patient. This algorithm was created by using the standard definition of the UPC Index and adapting it to the primary care health system in Québec (16). If they were to determined to have no primary care through Table 5, then the child’s index score was 0. Once each child’s individual UPC Index score was determine, each child was then categorized into one of the following tertiles; >0-0.4=low, >0.40-0.70=medium, and >0.70-1= high.

**Table 7 Algorithm for determining UPC Index score**

| **Algorithm to identify the UPC Index score** |
| --- |
| **STEP 1**  The patient’s assigned UPC should be determined first with the algorithm in Table 5. Once their UPC has been determined, the total amount of visit with their determined primary care model will be collected between January 1 2010 and December 31, 2011.  Family physician (FMG or non-FMG) is assigned for “primary care model”: count all visits with any billing code  Pediatrician is assigned for “primary care model”: count all visits with any billing code |
| **STEP 2**  The total amount of primary care visits billed with the patient will be collected between January 1, 2010 and December 31, 2011. Add up all visits with a family physician (any type of visit) plus PRIMARY CARE VISITS with pediatricians (use pediatrician codes in Table 9). The denominator will also include all the visits that were “counted” in STEP 1 (that have not been already “counted” i.e. no repeated visits). |
| **STEP 3**  The value determined in STEP 1 will be divided by the value determined in STEP 2 in order to give a ratio value that should range from 0 to 1. This value is their index score. |
| **STEP 4**  If no UPC is identified though in Table 5, then the subject does not have a UPC and their index score is 0. |

**Table 8 Primary care visit codes**

| **Description** | **Cabinet, CLSC, UMF-CH** | **CHSGS (external clinic)** |
| --- | --- | --- |
| Patient  ordinary exam <60 years | 08870 | 00005 |
| Patient  complete exam <60 years | 08871 | 00056 |
| Patient  major complete exam <60 years | 08872 | 00097 |
| Home visit <70 years, first patient, non-urgent | 00002 | Same (home) |
| Home visit, additional patients, ordinary exam | 08873 | Same (home) |
| Home visit, additional patients, complete exam | 08874 | Same (home) |
| Home visit, additional patients, complete psychiatric exam | 08855 | Same (home) |
| Home visit, loss of autonomy, first patient, all other times than 0-7h | 00007 |  |
| Home visit, loss of autonomy, additional patients | 00075 |  |
| Exam/Pregnancy “responsible for care” | 00059 | 00059 |
| Psychiatric complete | 08901 | 08807 |
| Psychiatric complete major | 08902 | 08809 |

**Table 9 Primary Care visits ONLY for Pediatricians**

| **Code** | **Details** |
| --- | --- |
| **OFFICE** | |
| 09194 | General exam in office by pediatrician |
| 09127 | Main (non-consultative) visit in office by pediatrician |
| 09129 | Follow-up visit by pediatrician |
| 15164 | Multidisciplinary or parent meeting in regards to a complex pathology |
| **HOME VISIT** | |
| 09171 | Main visit by pediatrician |
| 09172 | Follow-up visit by pediatrician |
| 15552 | Palliative care visit by pediatrician |

### Outcomes

Two outcome variables were created for this project; asthma-related ED visits and asthma-related hospital admissions. These two variables were measured during the outcome period of January 1,2012 to December 31, 2013. The establishment code 0X7 was used to identify ED visits that occurred during this outcome period. The ICD-9 and ICD-10 Codes in Table 10 were to differentiate those asthma-related ED visits from the rest. These codes were determined to be asthma-related based on a previous project that looked to develop an index of asthma control specific to children and adolescents and the investigators of this project determined these ICD codes to be asthma-related based on a definition of asthma control from the Canadian Thoracic Society 2012 Asthma Guidelines (18). A binary variable that separate those children who had at least one asthma-related ED from those who did not have a asthma related ED visit was created. The asthma related hospital admissions variable was created with the use of Med-Echo services during the outcome period of January 1,2012 to December 31,2013. The ICD codes in Table 10 were also used to determine those hospital admissions which were asthma-related. A binary variable was created which determined those children who had an asthma related hospital admission versus those children who did not.

**Table 10 Asthma-related ICD codes**

| **ICD 9 Code** | **ICD 10 Code** | **Details** |
| --- | --- | --- |
| 493xx | J45X, J46X | Asthma |
| 786.0 | R06.2 | Wheezing |
| 466xx | J20X, J21X | Acute Bronchitis and Bronchiolitis |
| 490 | J40X | Bronchitis not specified as acute or chronic |
| 519.1 | J98.0 | Acute Bronchospasm |

### Sensitivity Analyses

Sensitivity analyses were completed to ensure findings were not sensitive to the choice of metric and to test the association of coordination of care. The UPC Index was replaced with the Bice-Boxerman Continuity of Care (COC) Index A child’s COC Index score was determining by looking only primary visits made with primary care providers between January 1,2010 and December 31, 2011. The algorithm presented in Table 11 was used to determine the COC Index score for each patient. This algorithm was created by using the standard definition of the COC Index created by Bice created in 1977 (22). The score should give a value between 0 to 1. If they were to be determined to have no primary care through Table 5, then the child’s COC Index score was 0. Once each child’s individual COC Index score was determine, each child was then categorized into one of the following categories; >0-0.4=low, >0.40-0.70=medium, and >0.70-1= high.

**Table 11 Algorithm for determining COC Index using primary care visits and primary care providers**

| **Algorithm to identify the COC Index score** |
| --- |
| **STEP 1**  Determine the number of visits to each unique provider. Ex. A patient visits their UPC= 7 times, pediatrician#1= 5 times, and pediatrician#2=2 times, etc. |
| **STEP 2**  The total amount of primary care visits (including primary care, pediatrician, etc. visits in Table 15 and Table 16, along with asthma specialist visit) billed with the patient will be collected between January 1, 2010 and December 31, 2011. The denominator will also include all the visits that were “counted” in STEP 1 (that have not been already “counted” i.e. no repeated visits). |
| **STEP 3**  The following equation shown below will be used to find each patient’s index score. “n_i_” is the number of visits to each provider, which was determined in STEP 1. The value found in STEP 2 is “n” in the equation.    Ex. If the patient had visits with their UPC 7 times, a pediatrician 5 times, and a different provider 2 times. “n” would be 14. The score would look like this. (((7^2^)+ (5^2^) + (2^2^)) – 14))/ (14(14-1)) = ((49+25+4)-14)/182 = 64/182= 0.351.  This score should never exceed 1, because each of the visits with the different provider should be counted for in STEP 1. |
| **STEP 4**  If no UPC is identified though in Table 5, then the subject does not have a UPC and their index score is 0. |

**Appendix 2 Results from the Sensitivity Analyses**

**Table 12 Baseline characteristics of cohort by COC Index**

| **VARIABLES** | **Level of Continuity of Care by COC Index** | | | **ALL**  **N (%)** |
| --- | --- | --- | --- | --- |
|  | **Low Continuity of Care (>0-0.40)**  **N (%)** | **Medium Continuity of Care (>0.40-0.70)**  **N (%)** | **High Continuity of Care( >0.70)**  **N (%)** |  |
| **TOTAL** | 18,722 (57.6) | 4,898 (15.1) | 8,873 (27.3) | 32,493 (100.0) |
| **PRIMARY CARE MODEL** |  |  |  |  |
| Pediatrician | 6,567 (35.1) | 2,862 (58.4) | 4,314 (48.6) | 13,743 (42.3) |
| FMG | 6,630 (35.4) | 786 (16.1) | 2,048 (23.1) | 9,464 (29.1) |
| Non-FMG | 5,525 (29.5) | 1,250 (25.5) | 2,511 (28.3) | 9,286 (28.6) |
| **AGE CATEGORY** |  |  |  |  |
| 2-5 years old | 8,677 (46.3) | 2,499 (51.0) | 3,128 (35.2) | 14,304 (44.0) |
| 6-9 years old | 5,188 (27.7) | 1,286 (26.3) | 2,498 (28.1) | 8,972 (27.6) |
| 10-12 years old | 2,447 (13.1) | 582 (11.9) | 1,553 (17.5) | 4,582 (14.1) |
| 13-16 years old | 2,410 (12.9) | 531 (10.8) | 1,694 (19.1) | 4,635 (14.2) |
| **SEX** |  |  |  |  |
| Female | 7,580 (40.5) | 1,946 (39.7) | 3,552 (40.0) | 13,078 (40.2) |
| **SES** |  |  |  |  |
| Q1 (least deprived) | 4,627 (24.7) | 1,297 (26.5) | 1,988 (22.4) | 7,912 (24.3) |
| Q2 | 4,392 (23.5) | 1,115 (22.8) | 1,888 (21.3) | 7,395 (22.8) |
| Q3 | 3,331 (17.8) | 819 (16.7) | 1,620 (18.3) | 5,770 (17.8) |
| Q4 | 2,871 (15.3) | 761 (15.5) | 1,496 (16.9) | 5,128 (15.8) |
| Q5 (most deprived) | 2,848 (15.2) | 770 (15.7) | 1,546 (17.4) | 5,164 (15.9) |
| Missing | 653 (3.5) | 136 (2.8) | 335 (3.8) | 1124 (3.5) |
| **RURALITY** |  |  |  |  |
| Urban (population >100k) | 14,204 (75.9) | 3,917 (80.0) | 5,831 (65.7) | 23,952 (73.7) |
| Small cities (population 10k-100k) | 1,919 (10.3) | 395 (8.1) | 1,178 (13.3) | 3,492 (10.8) |
| Rural (population <10k) | 2,466 (13.2) | 562 (11.5) | 1,799 (20.3) | 4,827 (14.9) |
| Missing | 133 (0.7) | 24 (0.5) | 65 (0.7) | 222 (0.70) |
| **OTHER CO-MORBIDITIES** |  |  |  |  |
| Asthma Only | 17,087 (91.3) | 4445 (90.8) | 8152 (91.9) | 29,683 (91.4) |
| Asthma and other chronic diseases | 1,635 (8.7) | 453 (9.2) | 721 (8.1) | 2,809 (8.7) |
| **PREVIOUS ED VISITS** |  |  |  |  |
| 0 Visit | 7,107 (38.0) | 1,946 (39.7) | 3,625 (40.9) | 12,678 (39.0) |
| 1 Visit | 3,733 (20.0) | 1,078 (22.0) | 1,740 (19.6) | 6,551 (20.1) |
| 2-3 Visits | 4,172 (22.3) | 973 (19.9) | 1,865 (21.0) | 7,010 (21.6) |
| Over 4 Visits | 3,710 (19.8) | 901 (18.4) | 1,643 (18.5) | 1,643 (19.3) |
| **PREVIOUS HOSPITAL ADMISSION** |  |  |  |  |
| Yes | 6020 (32.1) | 1,341 (27.4) | 2,345 (26.4) | 9,706 (30.0) |
| **PREVIOUS ASTHMA SPECIALIST VISITS** |  |  |  |  |
| 0 Visit | 6,628 (35.4) | 1,173 (24.0) | 2,808 (31.7) | 10,609 (32.7) |
| 1 Visit | 3,083 (16.5) | 570 (11.6) | 963 (10.9) | 4,616 (14.2) |
| 2 Visits | 4,409 (23.6) | 1,394 (28.5) | 2,590 (29.2) | 8,393 (25.8) |
| Over 3 Visits | 4,602 (24.6) | 1,761 (36.0 | 2,512 (28.3) | 8,875 (27.3) |

CMC: Children with Medical Complexity; ED: Emergency Department; SES: Socioeconomic status; Q: Socioeconomic Quintile; FMG: Family Medicine Groups; UPC: Usual Provider of Care; IQR: Interquartile Range

**Table 12 Crude proportions and adjusted odds ratio of asthma-related acute outcomes**

| **Characteristics** | **No. (%) of children with an ED visit, by characteristic** | **ED visits,**  **OR (95% CI)** | **No. (%) of children with a hospital admission, by characteristic** | **Hospital admission, OR (95% CI)** |
| --- | --- | --- | --- | --- |
| **COC INDEX** |  |  |  |  |
| Low (>0-0.40) | 1,874/18722 (10.0) | Reference | 1120/18722 (6.0) | Reference |
| Medium (>0.40-0.70) | 429/4898 (8.8) | 0.89 (0.79, 1.00) | 237/4898 (4.8) | 0.84 (0.72, 0.98) |
| High (>0.70) | 937/8873 (10.6) | 1.10 (1.01, 1.21) | 490/8873 (5.5) | 0.99 (0.88, 1.11) |
| **PRIMARY CARE MODEL** |  |  |  |  |
| FMG | 961/9,464 (10.1) | Reference | 618/9,464 (6.5) | Reference |
| Pediatrician | 1,225/13,743 (8.9) | 0.97 (0.87, 1.07) | 643/13,743 (4.7) | 0.83 (0.72, 0.95) |
| Non-FMGs | 1,054/9,286 (11.3) | 1.11 (1.00, 1.22) | 586/9,286 (6.3) | 0.94 (0.83, 1.07) |
| **AGE CATEGORY** |  |  |  |  |
| 2-5 yo | 1,765/14,304 (12.3) | Reference | 1,041/14,304 (7.3) | Reference |
| 6-9yo | 789/8,972 (8.8) | 1.09 (1.00, 1.20) | 407/8,972 (4.5) | 0.94 (0.83, 1.07) |
| 10-12yo | 376/4,582 (8.2) | 1.20 (1.06, 1.37) | 192/4,582 (4.2) | 0.93 (0.79, 1.080) |
| 13-16yo | 310/4,635 (6.7) | 0.94 (0.82, 1.07) | 207/4,635 (4.5) | 0.91 (0.77, 1.08) |
| **SEX** |  |  |  |  |
| Female | 1,153/13,078 (8.8) | Reference | 724/13,078 (5.5) | Reference |
| Male | 2,087/19,415 (10.7) | 1.13 (1.04, 1.22) | 1,123/19,415 (5.8) | 0.97 (0.88, 1.07) |
| **SES** |  |  |  |  |
| Q1 (least deprived) | 613/7,912 (7.8) | Reference | 382/7,912 (4.8) | Reference |
| Q2 | 688/7,395 (9.3) | 1.07 (0.96, 1.20) | 422/7,395 (5.7) | 1.04 (0.90, 1.20) |
| Q3 | 585/5,770 (10.1) | 1.11 (0.99, 1.26) | 361/5,770 (6.3) | 1.11 (0.95, 1.29) |
| Q4 | 597/5,128 (11.6) | 1.27 (1.13, 1.44) | 300/5,128 (5.8) | 1.05 (0.89, 1.23) |
| Q5 (most deprived) | 638/5,164 (12.4) | 1.30 (1.16, 1.47) | 322/5,164 (6.2) | 1.08 (0.92, 1.26) |
| **RURALITY** |  |  |  |  |
| Urban (population >100k) | 2,230/23,952 (9.3) | Reference | 1,190/23,952 (5.0) | Reference |
| Small cities (population 10k-100k) | 391/3,492 (11.2) | 0.97 (0.86, 1.10) | 290/3,492 (8.3) | 1.36 (1.17, 1.57) |
| Rural (population <10k) | 610/4,827 (12.6) | 1.05 (0.95, 1.17) | 360/4,827 (7.5) | 1.15 (1.00, 1.32) |
| **OTHER CO-MORBIDITIES** |  |  |  |  |
| Asthma Only | 2,901/29,684 (9.8) | Reference | 1,269/29,684 (4.3) | Reference |
| Asthma & other comorbidities | 339/2,809 (12.1) | 0.83 (0.73, 0.94) | 578/2,809 (20.6) | 3.95 (3.52, 4.44) |
| **PREVIOUS HOSPITAL ADMISSION** |  |  |  |  |
| No | 1,707/22,787 (7.5) | Reference | 710/22,787 (3.1) | Reference |
| Yes | 1,533/9,706 (15.8) | 1.07 (0.99, 1.17) | 1,137/9,706 (11.7) | 1.92 (1.71, 2.15) |
| **PREVIOUS ED VISITS** |  |  |  |  |
| 0 Visit | 353/12,678 (2.8) | Reference | 278/12,678 (2.2) | Reference |
| 1 Visit | 448/6,551 (6.8) | 2.48 (2.15, 2.87) | 256/6,551 (3.9) | 1.43 ( 1.20, 1.71) |
| 2-3 Visits | 857/7,010 (12.2) | 4.60 (4.03, 5.25) | 474/7,010 (6.8) | 2.17 (1.84, 2.55) |
| Over 4 visits | 1,582/6,254 (25.3) | 10.67 (9.34, 12.18) | 839/6,254 (13.4) | 3.45 (2.94, 4.05) |
| **PREVIOUS ASTHMA SPECIALIST VISITS** |  |  |  |  |
| 0 Visits | 880/10,609 (8.3) | Reference | 551/10,609 (5.2) | Reference |
| 1 Visit | 497/4,616 (10.8) | 1.16 (1.02, 1.31) | 261/4,616 (5.6) | 1.03 (0.87, 1.20) |
| 2 Visits | 611/8,393 (7.3) | 0.97 (0.86, 1.09) | 295/8,393 (3.5) | 0.90 (0.77, 1.05) |
| Over 3 Visits | 1,252/8,875 (14.1) | 1.50 (1.35, 1.67) | 740/8,875 (8.3) | 1.45 (1.27, 1.65) |

OR: Odds Ratio; CI: Confidence Interval; CMC: Children with Medical Complexity; ED: Emergency Department; SES: Socioeconomic status; Q: Socioeconomic Quintile; FMG: Family Medicine Groups; UPC: Usual Provider of Care
